# Alteration of serum high-mobility group protein 1 (HMGB1) levels in children with enterovirus 71-induced hand, foot, and mouth disease

**DOI:** 10.1097/MD.0000000000006764

**Published:** 2017-04-28

**Authors:** Weikun Zheng, Haifan Shi, Yiping Chen, Zhiwei Xu, Jie Chen, Longteng Jin

**Affiliations:** Department of Childhood Infectious Diseases, The Second Affiliated Hospital and Yuying Children's Hospital of Wenzhou Medical University, Wenzhou, Zhejiang, China.

**Keywords:** hand, foot, and mouth disease, Enterovirus 71, high-mobility group box 1, IL-6, TNF-α

## Abstract

Hand, foot, and mouth disease (HFMD) is a common pediatric disease caused by enterovirus infection. It typically presents as a fever along with flat, discolored spots and bumps on the hands, feet, and mouth. Compared with other viruses, enterovirus 71 (EV71)-induced HFMD is more prone to cause severe complications in children, such as brainstem encephalitis, cardiopulmonary disorders, and even death. More in-depth studies are still necessary to understand the characteristics of EV71-induced HFMD, although some related research has been reported so far. High-mobility group box 1 (HMGB1) is an inflammatory cytokine that can upregulate other inflammatory factors through its receptors, such as Toll-like receptors and the receptor for advanced glycation endproducts.

We prospectively investigated the alteration of serum HMGB1, interleukin (IL)-6, and tumor necrosis factor (TNF)-α levels before and after treatment in 82 children with HFMD.

We found that the serum HMGB1, IL-6, and TNF-α levels were significantly increased in EV71-induced HFMD, and that these changes were more serious in the severe and critical HMFD groups; however, there was no significant difference in the HMGB1 level between the normal control and mild HMFD groups. Moreover, the serum HMGB1 level was positively correlated with the alteration of serum IL-6 and TNF-α concentrations.

These results suggest that HMGB1 is involved in the inflammatory pathogenesis of EV71-induced HFMD and that the serum level of HMGB1 could be applied as a clinical indicator for the severity of HFMD, and also a sign for the recovery prognosis of HFMD.

## 1 Introduction

Hand, foot, and mouth disease (HFMD) is a common pediatric disease caused by enterovirus infection. Currently, HFMD is frequently found in the Asia-Pacific region, especially China. Clinically, it typically presents with mild symptoms, such as a fever along with flat, discolored spots, and bumps on the hands, feet, and mouth. In some patients with severe disease, it normally is accompanied with several neurological complications (including cephalomeningitis, encephalitis, and neurogenic pneumonedema) and circulatory disorders. Occasionally, it even causes death.^[1]^

Enteroviruses are categorized into coxsackie group A (types 1–22, 24), coxsackie group B (types 1–6), echoviruses (types 1–7, 9, 11–27, 29–34), and enteroviruses (types 68–71). There are 4 species of human enteroviruses (HEV), including HEV-A, HEV-B, HEV-C, and HEV-D. Enterovirus 71 (EV71) belongs to the HEV-A species.^[2]^ EV71 and coxsakievirus A16 (CVA16) are the major causative agents of HFMD, although other strains of coxsackievirus and enterovirus, including CVA4-10, CVA24, coxsakievirus B2-5 (CVB2-5), and echovirus 18 (ECHO18), have been reported to cause HFMD.^[1]^ Compared with other viruses, EV71-induced HFMD is more prone to cause severe complications in children, such as brainstem encephalitis, cardiopulmonary disorders, and even death.^[1]^ Some neurological complications of EX71 infection present as an acute disorder of motor neurons such as weakness of the extremities, similar to poliomyelitis.^[1]^ Therefore, an early indicator of EV71 infection with neurological involvement is crucial for appropriate management.^[3]^ Almost every 3 years in the Asia-Pacific region, from 2000 to 2009, there were HFMD outbreaks.^[1]^ Due to the potential of EV71 infection causing severe complications, deeper and broader studies are still necessary to understand the characteristics of EV71-induced HFMD, although some related research has been reported so far. In addition, the pathogenic mechanism of HFMD is not deeply understood. However, some proinflammatory factors in the serum or central cerebrospinal fluid have been reported to be significantly increased in EV71-induced HFMD, including interleukin (IL)-4, IL-5, IL-22, IL-23, IL-2, tumor necrosis factor (TNF)-α, IL-1β,^[1]^ and IL-6.^[4]^ These findings indicate that the inflammatory process is involved in the pathogenesis of HFMD.

High-mobility group box 1 (HMGB1) is preliminarily reported as a DNA-binding protein. It facilities gene transcription through interactions with several transcription factors. HMGB1 can remodel chromatin through interactions with nucleosomes to relax the packed DNA.^[5]^ Based on the above mechanism, HMGB1 participates in some pathological processes, including cardiovascular disorders, tumor development, and ischemia/reperfusion damage.^[6,7]^ The innate immune responses can trigger its activity to partially modulate the immune state^[8]^ through binding to receptors, such as Toll-like receptor (TLR)2/TLR4, and the receptor for advanced glycation endproducts (RAGE), to upregulate inflammatory factors.^[9]^ TLRs and RAGE are classified into the pattern recognition receptor family and participate in the innate immune response.^[1]^ As pathogen-associated molecular patterns, TLRs can sense distinct exogenous external molecular products, whereas RAGE mostly recognizes endogenous molecules.^[7]^ Therefore, in the past decade, the HMGB1 activity in inflammatory disorders, including infectious and noninfectious diseases, has been paid increasing attention. Recombinant HMGB1 has been shown to induce IL-6 expression through RAGE, but not TLR2 or TLR4, during a fat inflammatory process in the human preadipocyte cell line SW872.^[1]^ In addition, TNF-α production has been shown to depend on HMGB1 in anoxia-reoxygenated cardiac myocytes.^[1]^

Based on the previous findings that the inflammatory process is related to the pathogenesis of HFMD, the activity of HMGB1 in HFMD has not yet been explored, and IL-6 and TNF-α are regulated in HFMD, we designed a prospective study to investigate the alteration of serum HMGB1, IL-6, and TNF-α levels before and after treatment of 82 children with EV71-induced HFMD. The results revealed a relationship between the serum concentrations of HMGB1, IL-6, and TNF-α and EV71-induced HFMD, and indicate that HMGB1 is involved in the pathogenesis of EV71-induced HFMD, suggesting that the gene transcription regulation by HMGB1 may play an impotent role during the process of HFMD.

## 2 Materials and methods

### 2.1 Patients

In all, 120 cases were selected for the preliminary study. Among them, 93 cases agreed to participate in the study, whereas 27 cases did not agree. Of these 93 cases, some patients were transferred to another hospital or discharged for some reasons; finally, 82 cases were enrolled. These 82 children who were suspected of having HFMD were hospitalized in the Department of Infectious Diseases, The Second Affiliated Hospital, and Yuying Children's Hospital of Wenzhou Medical University, Zhejian, China, from June 2014 to June 2015. Among them, 44 were boys and 38 were girls. The average age of the children was 26.0 months. The suspected patients were isolated in specific isolation wards. After confirmation with etiological detection by real-time polymerase chain reaction (PCR), the patients were symptomatically treated. Another 15 healthy infants hospitalized in the Department of Child Health Care for regular health examination during the same period were enrolled as the control group. This study was conducted with the approval of the Institutional Human Ethics Committee of Yuying Children's Hospital.

### 2.2 Diagnosis and group classification

All of the patients were diagnosed and evaluated according to the Guidelines for Diagnosis and Treatment of HFMD (2010 edited version) by the Ministry of Health of the People's Republic of China (http://www.moh.gov.cn/mohyzs/s3586/201004/46884.shtml), in which epidemic season, age of onset, fever, and typical flat, discolored spots and bumps on the hands, feet, mouth, and buttocks were the major factors considered. The enrolled patients were etiologically confirmed by EV71 real-time PCR. According to the clinical characteristics, these patients were divided into 3 groups: mild HFMD group with 30 children (13 girls, 17 boys), severe HFMD group with 40 patients (18 girls, 22 boys), and critical HFMD group with 12 patients (7 girls, 5 boys). In the mild HFMD group, the patients presented with a rash on their hands, feet, mouth, and buttocks with or without a fever. In the severe HFMD group, the patients showed symptoms of nervous system involvement, including depression, lethargy, easily frightened, delirium, headache, vomiting, limb tremors, myoclonus, nystagmus, ataxia, eye movement disorders, powerless or acute flaccid paralysis, and even convulsions. Signs of visible meningeal irritation, and also weakened or disappeared tendon reflexes were also observed. In the critical HFMD group, 1 of the following symptoms could also be seen: frequent convulsions, coma, brain hernia, dyspnea, cyanosis, bloody foamy sputum, pulmonary rales, shock, and other circulatory insufficiency.

### 2.3 Real-time PCR of EV71

Real-time PCR was performed with TaqMan technology to detect the EV71 genomic DNA in all throat swab samples collected from the HFMD patients. In brief, total RNA was extracted from throat swab specimens of the patients by a QIAamp viral RNA Mini Kit (Qiagen, Hilden, Germany), according to the manufacturer's protocol. Then, the EV71 gene was detected with a 1-step RT-PCR Detection Kit (Da An Gene, Guangzhou, China) and the 7500 real-time RTPCR system (Applied Biosystem, Foster City, CA) with the following program: 1 cycle of 40°C for 25 minutes, another cycle of 95°C for 3 minutes, and 40 cycles of 2-step PCR at 93°C for 15 seconds and 55°C for 45 seconds. Each sample assay was repeated twice. If outlier results were found, the samples were reassayed to confirm the findings. The averaged data were used for calculation.

### 2.4 Serum sample collection

The peripheral blood samples were collected at three different time points: the first day of hospitalization (TP0), improved condition (TP1), and recovered from disease (TP2). The TP1 time point was defined as the time that the mental state was significantly improved, the nervous system lesions were obviously reduced, the body temperature was below 38°C, and the white blood cell (WBC) count and blood sugar levels were notably decreased. The TP2 time point was defined as the time that all the nervous system lesions, cardiovascular and pulmonary function, body temperature, WBC count, and blood sugar levels had recovered to normal levels. The serum was isolated by collecting the supernatant of blood prewarmed to 37°C.

### 2.5 Enzyme-linked immunosorbent assay (ELISA) of serum HMGB1, IL-6, and TNF-α

The serum HMBG1 level was detected by an HMBG1 detection kit (Chondrex; Redmond, WA). The serum IL-6 and TNF-α levels were analyzed with a Human IL-6 Quantikine ELISA kit and a Human TNF-α Quantikine ELISA kit (R&D; Minneapolis, MN). In brief, 200 μL of sample was mixed with 50 μL of Assay Diluent RD1F to each well of the HMGB1, IL-6, or TNF-α ELISA plate and incubated for 2 hours at room temperature. After 3 washes with Wash Buffer (400 μL each), 200 μL of HMGB1, IL-6, or TNF-α conjugate was added to each well and incubated for 1 hour at room temperature. After 3 washes, 200 μL of Substrate Solution was added to each well, and the mixture was incubated for 20 minutes in the dark at room temperature. Finally, 50 μL of Stop Solution was added to each well to stop the reaction. The optical density (450 nm) of each well was determined within 30 minutes by a microplate reader (Biotek). The minimum detectable concentrations for the kits are 0.8 ng/mL for serum HMGB1, 10.7 pg/mL for serum IL-6, and 1.6 pg/mL for serum TNF-α. Each sample assay was repeated twice. If outlier results were found, the samples were reassayed to confirm the findings. The averaged data were used for calculation.

### 2.6 Statistical methods

All data were analyzed with SPSS18.0 software (SPSS Inc., Chicago, IL). The data were analyzed with the paired *t* test and repeated-measures analysis of variance, which showed that there was no correlation between the repeated measurement data. The measured data were in line with Huynh-Feldt conditions. The quantitated results were represented as the mean ± standard deviation (SD). The *t* test was applied for average data comparison of 2 groups. Analysis of variance was used to compare the averaged data of multiple groups. The correlative analysis between HMGB1 and IL-6 or TNF-α was performed with the Spearman rank correlation test. A 2-tailed *P* value less than .05 was considered as a significant difference.

## 3 Results

### 3.1 Clinical data

The EV71 infection of all the enrolled HFMD patients was verified by EV71 real-time PCR. In the 3 groups of the EV71-infected HFMD patients, the average age of the 30 patients (13 girls, 17 boys) enrolled in the mild HFMD group was 26.77 ± 9.49 months; the average age of the 40 patients (18 girls, 22 boys) enrolled in the severe HFMD group was 25.78 ± 8.08 months; and the average age of the 12 patients (7 girls, 5 boys) enrolled in the critical HFMD group was 25.17 ± 7.66 months. There was no significant difference in terms of sex or age among these 3 groups (*P* > .05). However, the symptoms of fever (above 39 °C for more than 3 days), depression, vomiting, easily frightened, limb tremors, shortness of breath, increased heart rate, capillary refilling time (>2 seconds), serum glucose (>8.3 mmol/L), and WBC count were all obviously different between groups, except the WBC between the normal controls and the mild or severe HFMD group (Table 1).

**Table 1:**
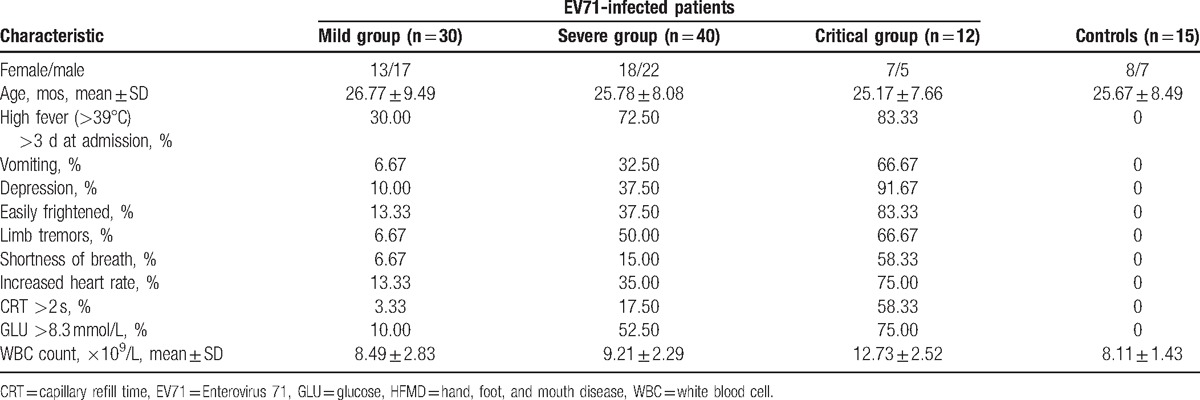
Clinical data of the HFMD patients and controls.

### 3.2 The serum HMGB1, IL-6, and TNF-α levels before treatment

**Figure 1:**
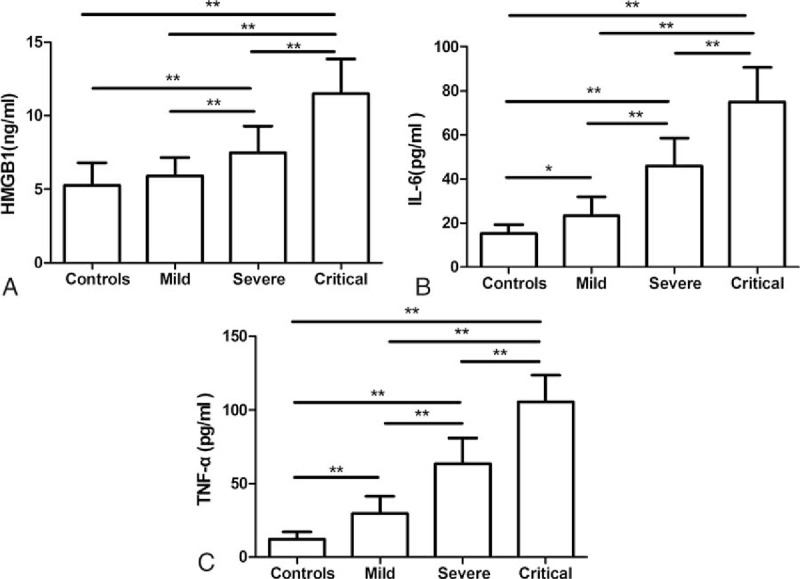
Serum HMGB1, IL-6, and TNF-α levels in EV71-infected HFMD patients and healthy controls. The serum HMGB1 (A), IL-6 (B), and TNF-α (C) levels were detected by ELISA in mild (n = 30), severe (n = 40), and critical (n = 12) cases of EV71-induced HFMD, and also normal controls (n = 15). The levels are represented as the mean ± SD. ∗*P* < .05, ∗∗*P* < .01. ELISA = enzyme-linked immunosorbent assay, EV71 = Enterovirus 71, HFMD = hand, foot, and mouth disease, HMGB1 = high-mobility group box 1, IL = interleukin, TNF = tumor necrosis factor.

The serum HMGB1, IL-6, and TNF-α levels of all the patients and controls were detected before treatment (the day of hospitalization) (TP0). The HMGB1 levels in the EV71-infected severe group (7.48 ± 1.80 ng/mL) and the critical HFMD group (11.50 ± 2.38 ng/mL) were significantly higher than those in the mild (5.91 ± 1.27 ng/mL) and control (5.28 ± 1.54 ng/mL) groups (*P* < .01). Besides, the HMGB1 level of the critical HFMD group was obviously higher than that of the severe HFMD group (*P* < .01). However, no difference was found between the mild HFMD and control groups (*P* > .05) (Fig. 1A). The levels of IL-6 (Fig. 1B) and TNF-α (Fig. 1C) showed similar alteration patterns as HMGB1 in that the serum levels were elevated along with the seriousness of the clinical situation. The IL-6 and TNF-α levels in the severe (46.01 ± 12.65 and 63.39 ± 17.54 pg/mL, respectively) and the critical HFMD (75.03 ± 15.69 and 105.59 ± 17.98 pg/mL, respectively) groups were significantly higher than those in the mild (23.34 ± 8.70 and 29.75 ± 11.64 pg/mL, respectively) and control (15.40 ± 3.89 and 12.33 ± 4.81 pg/mL, respectively) groups (*P* < .01–.05). The differences between the groups were significant as well (*P* < .01–.05).

### 3.3 The serum levels of HMGB1, IL-6, and TNF-α after treatment

**Figure 2:**
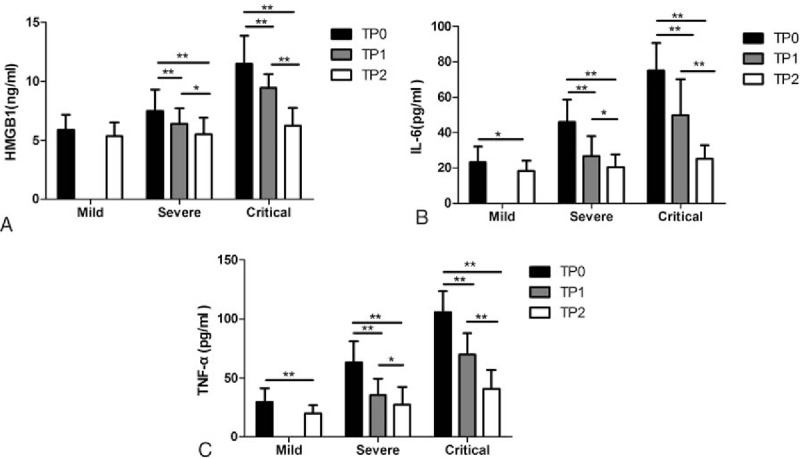
Serum HMGB1, IL-6, and TNF-α levels of EV71-infected HFMD patients at different clinical stages. The serum HMGB1 (A), IL-6 (B), and TNF-α (C) levels were examined by ELISA (mean ± SD) at 3 time points: first day of hospitalization (TP0, black), improved condition (TP1, gray), and disease recovery (TP2, white). The tested numbers in each group are the same as in Figure 1. The levels are represented as the mean ± SD. ∗*P* < .05, ∗∗*P* < .01. ELISA = enzyme-linked immunosorbent assay, EV71 = Enterovirus 71, HFMD = hand, foot, and mouth disease, HMGB1 = high-mobility group box 1, IL = interleukin, TNF = tumor necrosis factor.

After symptomatic treatment, the levels of HMGB1, IL-6, and TNF-α were examined when the condition improved (TP1) and when the patient had recovered from disease (TP2), respectively. Interestingly, all the abnormal levels of HMGB1, IL-6, and TNF-α were notably decreased after treatment. Except for the HMGB1 level in the mild group, which did not show a significant difference between before (T0) and after (T2) treatment (Fig. 2A), the levels of HMGB1 (Fig. 2A) were obviously decreased from T0 (critical group: 11.50 ± 2.38 ng/mL; severe group: 7.45 ± 1.80 ng/mL) to T1 (critical group: 9.46 ± 1.15 ng/mL; severe group: 6.39 ± 1.34 ng/mL), and to T2 (critical group: 6.27 ± 1.47 ng/mL; severe group: 5.53 ± 1.40 ng/mL) (*P* < .01–.05). In addition, the serum IL-6 levels (Fig. 2B) were significantly decreased from T0 (critical group: 75.03 ± 15.69 pg/mL; severe group: 46.01 ± 12.65 pg/mL; mild group: 23.34 ± 8.70 pg/mL) to T1 (critical group: 49.76 ± 20.56 pg/mL; severe group: 26.59 ± 11.47 pg/mL) and to T2 (critical group: 25.16 ± 7.68 pg/mL; severe group: 20.44 ± 7.38 pg/mL; mild group: 18.14 ± 6.08 pg/mL) (*P* < .01–.05). Similarly, the serum TNF-α levels (Fig. 2C) were considerably decreased from T0 (critical group: 105.59 ± 17.98 pg/mL; severe group: 63.39 ± 17.54 pg/mL; mild group: 29.75 ± 11.64 pg/mL) to T1 (critical group: 69.76 ± 18.33 pg/mL; severe group: 35.62 ± 13.64 pg/mL) and to T2 (critical group: 41.02 ± 15.89 pg/mL; severe group: 27.43 ± 14.84 pg/mL; mild group: 19.88 ± 7.15 pg/mL) (*P* < .01–.05). The blood sample collection at T1 in the mild group was not performed; therefore, the corresponding data are not available.

### 3.4 Correlation analysis of the serum HMGB1 level to the serum IL-6 or TNF-α level

**Figure 3:**
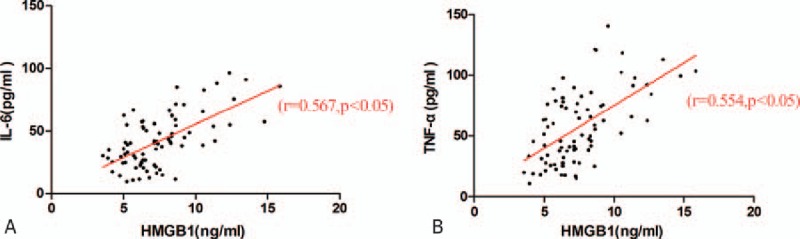
Correlation analysis of the serum IL-6 and TNF-α levels with the HMGB1 levels. The correlation between serum HMGB1 and IL-6 (A) or TNF-α (B) was analyzed with the Spearman rank correlation test among all the samples, respectively. The corresponding correlation coefficient (*r)* and *P* value are indicated in the graphs. HMGB1 = high-mobility group box 1, IL = interleukin, TNF = tumor necrosis factor.

The correlation of the serum levels of HMGB1 and IL-6 was analyzed with Spearman rank-order correlation (Fig. 3A). The correlation coefficient, *r*, of HMGB1/IL-6, was 0.567, which indicated that the alteration of the serum HMGB1 level was positively correlated to the serum IL-6 level. Additionally, the serum level of HMGB1 was shown to be positively correlated to the serum TNF-α level (*r* = 0.554) by Spearman rank-order correlation (Fig. 3B).

## 4 Discussion

Hand, foot, and mouth disease is a childhood acute disease caused by enterovirus infection. Severe complications (such as brain stem encephalitis, neurogenic pulmonary edema, and other fatal complications) and a high mortality due to HFMD are more frequently related to EV71 infection.^[10,11]^ In response to cellular stress, as a major factor of innate immune responses and a partial regulator of adaptive immunity,^[8]^ HMGB1 is passively released into the extracellular environment during cell death, and also actively secreted by mononuclear cells and other cell types.^[7]^ After binding to its receptors (such as RAGE and TLRs), HMGB1 causes the release of inflammatory cytokines and chemokines, and also the expression of the corresponding receptors.^[9]^ In this study, we observed that the serum HMGB1 level increased depending on the severity of viral infection during the process of EV71-induced HFMD among 82 children (Fig. 1A). This alteration almost paralleled with the variation of serum IL-6 and TNF-α, which are proinflammatory factors and are reported to be elevated in severe HFMD.^[1]^ Ooi et al have investigated several proinflammatory cytokines in HFMD and found that IL-6 and TNF-α are elevated in HFMD and that this elevation is consistent with the severity of disease.^[1]^ This pattern was verified in our observations, and also by the fact that the IL-6 and TNF-α level increases were correlated with the severity of HFMD in the mild, severe, and critical groups (Fig. 1). In addition, Wang et al^[12]^ have suggested that HMGB1 may have pathological potential in diseases caused by viral infection, since they found that HMGB1 is elevated in West Nile virus, Salmon anemia virus, and even enterovirus (Coxsackievirus B3)-infected patients.^[13]^ Nevertheless, whether HMGB1 participates in EV71-induced HFMD is still unclear. Here, we showed that EV71 infection triggers not only IL-6 and TNF-α secretion but also HMGB1 release. Different from the changes of serum IL-6 and TNF-α in EV71-induced HFMD, the serum HMGB1 elevation only occurred in the severe and the critical HFMD groups. In other words, there was no difference between the normal control group and the mild HFMD group (Fig. 1A). These results combined with a previous finding^[1]^ indicate that the responses of serum IL-6 and TNF-α are more sensitive or faster than that of serum HMGB1 in EV71-induced HFMD. This finding is consistent with the results of Wang et al that HMGB1 release occurs 16 to 32 hours after TNF-α, and IL-1 release during endotoxemia.^[14]^ On the contrary, this elevated HMGB1 level may provide a valuable indicator clinically to signal the severity of EV71-induced HFMD. The localization of HMGB1 determines its different activities. For example, intracellular HMGB1 has transcriptional activity through binding with chromatin structures of DNA. Alternatively, extracellular HMGB1 can be either actively secreted or passively released during the signaling of cell death 6 protein, which performs damage-associated molecular pattern (DAMP) activity through TLRs and RAGE to promote an immune response.^[15,16]^ The serum HMGB1 level should be classified into the extracellular form. Therefore, it may perform its inflammatory activity as DAMP activity through TLRs or RAGE.

Furthermore, the alteration of serum HMGB1, and also IL-6 and TNF-α levels was investigated before and after treatment of EV71-induced HFMD. After symptomatic treatment, the serum levels of HMGB1, IL-6, and TNF-α were gradually decreased from before treatment (T0) until the condition improved (TP1), and then to disease recovery (TP2) (Fig. 2). These results suggest that along with recovery from HFMD, the HMGB1, IL-6, and TNF-α levels decreased correspondingly, indicating that not only IL-6 and TNF-α but also HMGB1 could be an indicator to show the recovery prognosis of HFMD.

Proinflammatory cytokines, such as IL-1, IL-6, and TNF-α, are tremendously increased in the early stage of EV71-induced HFMD. The strong increase of the proinflammatory cytokines elevates the permeability of the blood–brain barrier and triggers the inflammatory cascade to produce more cytokines (over-response), which subsequently progresses into systemic inflammatory response syndrome, resulting in encephalitis, cardiopulmonary failure, and pulmonary edema.^[4,17]^ Here, we found that another proinflammatory cytokine, HMGB1, which abnormally increased along with the progression of EV71-induced HFMD, showed a highly positive correlation with IL-6 and TNF-α in the serum (Fig. 3). This finding indicates that HMGB1 may participate in the pathogenic process of EV71-induced HMFD through activating transcription of some inflammatory cytokines via TLR4 binding^[1]^ and interaction with nucleosomes to bind to DNA.^[5,18]^ The serum level of HMGB1 could be taken as an indicator for the severity of EV71-induced HMFD. Indeed, it also has been reported by others that the elevation of the HMGB1 level correlates with the severity of heart failure in patients.^[1]^ Furthermore, RAGE, the specific receptor of HMGB1, has been identified as a prognostic factor in human heart failure.^[1]^ In addition, an elevated HMGB1 level has been reported to correlate with the severity of cardiac disorders; thus, it has been suggested that an increased HMGB1 level may be used as a diagnostic parameter.^[1]^ Another study has shown that the serum levels of HMGB1 indicate the severity of disease in patients with HIV.^[19]^ Moreover, dengue virus can induce HMGB1 release from endothelial and dendritic cells, and the serum HMGB1 levels of patients with dengue fever are related to the clinical symptoms.^[20]^

The critical role of HMGB1 in many infectious and noninfectious inflammatory diseases has been described previously. Nosaka et al have reported that the plasma HMGB1 level significantly increased on the ninth day (the peak period of death) after viral infection in an influenza virus infection mouse model. Administration of an anti-HMGB1 monoclonal antibody prevented the inflammatory response and improved the survival rate of the mice.^[21]^ HMGB1 inhibition with glycyrrhizin pharmacologically or anti-HMGB1 antibody obviously decreased the myocardial inflammation induced by troponin I in mice.^[22]^ Other data also support that neutralization of HMGB1 by antibodies could defend against tissue injury in ischemia, rthritis, endotoxemia, sepsis, colitis, and systemic lupus erythematosus.^[8,14]^ In addition, inhibition of RAGE, the receptor of HMGB1, significantly decreases the levels of several inflammatory mediators (including chemokines, chemokine receptors, matrix metalloproteinases, and cytokines), which are critical pathogenic factors in autoimmune myocarditis.^[23]^ Moreover, nuclear factor-κB-binding activity, a mechanism of HMGB1 signaling,^[24]^ was reduced in immunized RAGE-knocked down mice.^[22]^ Therefore, whether neutralization of HMGB1 or its receptor (such as RAGE and TLRs) with the corresponding antibody or chemical could attenuate or ease EV71-induced HFMD would be valuable to test clinically in the future.

## 5 Conclusions

In this prospective study, we observed the alteration of serum HMGB1, IL-6, and TNF-α levels in 3 groups of EV71-infected HFMD patients with different severity levels before and after treatment. We found that the serum levels of HMGB1, IL-6, and TNF-α were significantly increased in the EV71-infected HFMD patients, and that these changes were more serious in the severe and critical groups; however, no significant difference in the HMGB1 levels was found between the normal control and mild HFMD group. Moreover, the serum HMGB1 level was positively correlated to the alteration of serum IL-6 and TNF-α concentrations. These results suggest that HMGB1 is involved in the inflammatory pathogenesis of HFMD, and that the serum level of HMGB1 could be applied as a clinical indicator for the severity of HFMD. In addition, the decrease of the HMGB1 level could be used as a sign for the recovery prognosis of HFMD.
